# Effect of sarcomere and mitochondria-related mutations on myocardial fibrosis in patients with hypertrophic cardiomyopathy

**DOI:** 10.1186/s12968-021-00718-3

**Published:** 2021-03-04

**Authors:** Hyemoon Chung, Yoonjung Kim, Chul-Hwan Park, Jong-Youn Kim, Pil-Ki Min, Young Won Yoon, Tae Hoon Kim, Byoung Kwon Lee, Bum-Kee Hong, Se-Joong Rim, Hyuck Moon Kwon, Kyung-A Lee, Eui-Young Choi

**Affiliations:** 1 Division of Cardiology, Department of Internal Medicine, Kyung Hee University School of Medicine, Seoul, South Korea; 2 Department of Laboratory Medicine, Gangnam Severance Hospital, Yonsei University College of Medicine, 211 Eonju-Ro, Gangnam-Gu, Seoul, 06273 Republic of Korea; 3 Department of Radiology, Gangnam Severance Hospital, Yonsei University College of Medicine, Seoul, South Korea; 4 Division of Cardiology, Heart Center, Gangnam Severance Hospital, Yonsei University College of Medicine, 211 Eonju-Ro, Gangnam-Gu, Seoul, 06273 Republic of Korea

**Keywords:** Hypertrophic cardiomyopathy, Myocardial fibrosis, Sarcomere gene mutation, Mitochondria

## Abstract

**Background:**

Myocardial fibrosis is an important prognostic factor in hypertrophic cardiomyopathy (HCM). However, the contribution from a wide spectrum of genetic mutations has not been well defined. We sought to investigate effect of sarcomere and mitochondria-related mutations on myocardial fibrosis in HCM.

**Methods:**

In 133 HCM patients, comprehensive genetic analysis was performed in 82 nuclear DNA (33 sarcomere-associated genes, 5 phenocopy genes, and 44 nuclear genes linked to mitochondrial cardiomyopathy) and 37 mitochondrial DNA. In all patients, cardiovascular magnetic resonance (CMR) was performed, including 16-segmental thickness, late gadolinium enhancement (LGE), native and post-T1, extracellular volume fraction (ECV), and T2, along with echo-Doppler evaluations.

**Results:**

Patients with sarcomere mutation (SM, n = 41) had higher LGE involved segment, % LGE mass, ECV and lower post-T1 compared to patients without SM (n = 92, all p < 0.05). When classified into, non-mutation (n = 67), only mitochondria-related mutation (MM, n = 24), only-SM (n = 36) and both SM and MM (n = 5) groups, only-SM group had higher ECV and LGE than the non-mutation group (all p < 0.05). In non-LGE-involved segments, ECV was significantly higher in patients with SM. Within non-SM group, patients with any sarcomere variants of uncertain significance had higher echocardiographic Doppler E/e’ (p < 0.05) and tendency of higher LGE amount and ECV (p > 0.05). However, MM group did not have significantly higher ECV or LGE amount than non-mutation group.

**Conclusions:**

SMs are significantly related to increase in myocardial fibrosis. Although, some HCM patients had pathogenic MMs, it was not associated with an increase in myocardial fibrosis.

## Background

Myocardial fibrosis, especially replacement fibrosis, is an important prognostic factor in hypertrophic cardiomyopathy (HCM) [1]. It causes lethal ventricular arrhythmia, exercise intolerance due to decreased ventricular compliance, atrial fibrillation, and progression to left ventricular (LV) systolic dysfunction. Although validated pathogenic sarcomere gene mutations (SMs) are the primary contributors to LV hypertrophy, a wide spectrum of genetic mutations, a sarcomere variant of uncertain significance (VUS) [2], and phenocopy gene and mitochondria-related mutations (MM) [3] also have been shown to be associated with HCM [4]. However, their degrees of contribution to myocardial fibrosis have yet to be extensively investigated.

Previous studies showed that pathogenic or likely pathogenic SMs are related to a higher prevalence and amount of LV fibrosis measured by cardiovascular magnetic resonance (CMR) late gadolinium enhancement (LGE) and native T1 mapping [5–8]. However, the results were controversial, especially in non-LGE segments, due to the limited number of patients, candidates of genetic mutations (e.g., only *MYBPC3* or *MYH7*), or techniques for myocardial tissue characterization. Recently, patients with sarcomere VUS were reported as having worse prognosis than the mutation negative patients [9]. Moreover, several basic science and translational studies suggest that MM (nuclear or mitochondrial DNA) are related to HCM and arrhythmic events [10]. We also recently published that MM were related to apical hypertrophy, a relatively benign phenotype, in a Korean population [11].

Regarding accurate myocardial tissue characterization, LGE is a validated method for replacement fibrosis. However, the pathological finding of HCM is complex and consists of myocyte disarray, diffuse or conglomerated replacement fibrosis, and intramyocardial small vessel fibrosis. Thus, LGE alone is not sufficient to show all pathological changes in HCM. With the recently developed T1/T2 mapping techniques conjoined with extracellular gadolinium distribution, CMR could make it possible to measure extracellular volume fraction (ECV) and the degree of myocardial inflammation. One of the strengths of the T1/T2 mapping technique is that it provides the degree of interstitial fibrosis and inflammation in the remote myocardium or gray zone in LGE imaging. Therefore, this technique would provide tissue characterization in healthy looking regional myocardium, in addition to the global LGE amount.

To overcome these limitations, we investigated the relationship between genetic mutations and myocardial tissue characteristics using extensive targeted genetic analysis in nuclear DNA (nDNA) and mitochondrial DNA (mtDNA). For accurate tissue characterization of LV myocardium, global and American Heart Association 16-segmental thickness, LGE amount, ECV, native T1, post-contrast T1, and T2 values were measured using CMR in HCM patients.

## Methods

### Study population

Of the 212 HCM patients who were enrolled in genetic study [11], 133 underwent CMR with LGE and T1/T2 mapping. The patients enrolled in the study had maximal LV hypertrophy greater than 13 mm and a ratio of maximal thickness to inferolateral wall thickness greater than 1.3 without an underlying cause of hypertrophy, such as uncontrolled hypertension or aortic stenosis. Both the pure apical type (hypertrophy confined below the papillary muscle level) and the mixed type (apical hypertrophy combined with asymmetrical septal hypertrophy at the mid-LV level, but with maximal thickness in the apex) were categorized as apical HCM [12]. All patients underwent screening analysis for Anderson-Fabry disease and were confirmed negative for the galactosidase alpha variant. The study protocol was approved by our institutional review board (3-2015-0019), and written informed consent was obtained from each participant.

### Genetic testing and analysis

#### HCM gene panel design for nDNA and mtDNA

A literature search of the PubMed database was performed to select targeted genes for the comprehensive HCM-specific gene panel, and 82 nDNA genes were included: 33 sarcomere protein genes, 5 phenocopy genes, and 44 nuclear genes linked to mitochondrial cardiomyopathy. HCM genes consisted of 8 validated sarcomere genes and 25 putative HCM genes [4, 11].

#### DNA preparation, library construction and sequencing of the HCM gene panel and mtDNA

##### Data analysis of the HCM gene panel and mitochondrial genome [11]

The details are described in Additional file 1: Method S1.

#### Classification of pathogenic/likely pathogenic variants and VUS

For 33 HCM genes, annotated variants using ANNOVAR and Variant Effect Predictor were classified as pathogenic, likely pathogenic, VUS, likely benign or benign based on refined American College of Medical Genetics and Genomics (ACMG) standards and guidelines for inherited cardiac conditions [13]. For 44 mitochondria-related nDNA genes (recessive conditions), annotated variants were classified as pathogenic and likely pathogenic based on ACMG guidelines. And we adapted gnomAD AF cutoff 0.01% as the moderate level of evidence supporting pathogenicity (ACMG/AMP criterion PM2) based on maximum credible population AF [13].

#### Identification of potential pathogenic mtDNA variants

Non-haplogroup-associated novel and rare variants were evaluated for potential pathogenicity based on variant location, amino acid change, and evolutionary conservation [14]. We interpreted mitochondrial variants using mitochondrial genome databases. We have assessed potential pathogenicity of novel and rare non-haplogroup-associated variants using multiple software programs including Polyphen2, Fathmmw, Mutation Assessor, and PROVEAN. When the majority of computational evidence supported a deleterious effect, we have assigned novel and rare non-haplogroup-associated variants as damaging mtDNA variants. Clinically relevant variants in mitochondrial genome databases and probably damaging nonsynonymous mtDNA variants in silico prediction were considered damaging mtDNA variants. The mitochondria-related deleterious variations (MM) consisted of damaging variants of mtDNA and likely pathogenic/pathogenic mutations of mitochondrial-nDNA. [11]. To evaluate systemic involvement in mitochondrial dysfunction, 19 questions were answered by all subjects. Detailed questions are described in Additional file 1: Method S2.

### Echocardiographic analysis

The details are described in Additional file 1: Method S3.

### Cardiovascular magnetic resonance imaging

CMR was performed using a 1.5-T CMR scanner (Magnetom Avanto; Siemens Healthineers, Erlangen, Germany) with a phased array body coil. The LV 2-, 3-, 4-chamber, and short-axis views were obtained using cine images with balanced steady-state free precession (bSSFP) sequence. LGE imaging was obtained 10 min after injection after administration of a gadolinium-based contrast agent (0.2 mmol/kg gadoterate dimeglumine; Dotarem, Guerbet, Paris, FR) with a fast gradient echo sequence prepared with magnitude- and phase-sensitive inversion recovery (PSIR). A bolus of contrast media was intravenously administered at 2 mL/s, followed by 20 mL normal saline at 4 mL/s through a 20-gauge cannula in the antecubital vein using a power injector (Nemoto; Nemoto Kyorindo, Tokyo, Japan). The appropriate inversion time before LGE imaging was determined using a fast gradient echo sequence with varied inversion times (150–650 ms) to null the signal from the normal myocardium. The following LGE imaging parameters were used: TR, 8.8 ms; TE, 3.36 ms; flip angle, 25°; acquisition matrix, 256 $\times$ 166; and field of view, 276 $\times$ 340 mm. Native T1 mapping with a modified Look-Locker inversion recovery (MOLLI) technique was performed during the mid-diastolic phase, and post-T1 mapping was performed 15 min after contrast media injection using the same slice axis and parameters as the pre-T1 mapping [15]. Quantitative T2 mapping imaging was performed before contrast media injection with a T2-prepared bSSFP pulse sequence along the same short-axis planes used for cine imaging. A motion correction algorithm provided by the vendor was used to reduce motion artifacts. The following acquisition parameters were used for T2 mapping: T2 preparation times, 0, 24, and 55 ms; TR, 3 $\times$ R-R ms; acquisition matrix, 126 $\times$ 192; acquisition time, 7 $\times$ R-R; single-shot acquisition; flip angle, 70°; and bandwidth, 916 Hz/pixel. T2-pixel maps were generated after motion correction using commercially available software on the scanner's workstation (Syngo; Siemens Healthineers) [16]. The LV was divided into 16 regional segments according to American Heart Association guidelines, and the average thickness within each segment was measured [17]. In the regional analysis, the anteroseptum was defined as segments 1, 2, 3, 7, 8, 9, 13, and 14; septum, as 2, 3, 8, 9, and 14; inferoposterior segment, as 4, 5, 10, 11, and 15; lateral segment, as 6, 11, and 16; and apical segment, as 13, 14, 15, and 16.

#### Measurement of late gadolinium enhancement

The presence of LGE involvement in each segment and the total number of LGE-involving segments were determined. In addition, the pattern of LGE and the percentage of LGE in LV mass were measured using dedicated quantitative analysis software (QmassMR 7.5 or 8.1, Medis Medical Imaging, Leiden, The Netherland) on PSIR LGE images [16]. To improve the reproducibility, a radiologist and a cardiologist, each with more than 10 years of experience analyzed LGE data. In each short-axis slice image, boundaries of contrast-enhanced areas were automatically traced. On LGE-CMR images, myocardium with abnormal enhancement was defined as an area of hyperenhancement more than 5 standard deviations from the remote myocardium. Remote myocardium was defined as nonenhanced myocardium, the opposite of hyperenhanced myocardium [18]. The maximal signal was determined by computer-assisted window thresholding of the enhanced area. Obvious artifacts, such as those caused by motion, were excluded using a tool from the software package. Total LGE volume was calculated by summing the LGE volumes of all the slices [19].

#### Measurement of native T1, extracellular volume fraction, and T2

With QMap and QECV-RE (Medis Medical Imaging), T2, native T1 (n = 128), post-T1, and ECV (n = 125) analyses were performed in the 128 patients. The myocardial ECV was automatically calculated with the following equation:

$${\text{ECV}} = (\Delta {\text{R1}}\;{\text{of}}\;{\text{myocardium}}/\Delta {\text{R1}}\;{\text{of}}\;{\text{LV}}\;{\text{blood}}\;{\text{pool}}) \times (1 - {\text{hematocrit}}),$$

where R1 = 1 / T1 and $\Delta$ R1 = post-contrast R1 $-$ pre-contrast R1 [20].

For normal control, we enrolled four healthy subjects and analyzed their ECVs. As a positive control, ECVs were analyzed in eight subjects who underwent CMR with T1 mapping for the evaluation of the cause of aborted sudden cardiac death or idiopathic ventricular tachycardia but had normal LV systolic function and structure both on echocardiography and CMR.

### Statistical analysis

Continuous variables with normal distributions are reported as the mean ± standard deviation or 95% confidence intervals. Student’s *t*-tests were used to compare the means of continuous variables that were approximately normally distributed between the two groups. Normality was determined using the Shapiro–Wilk test. Categorical variables are reported as counts (or percentages) and were compared using chi-square tests. For comparisons of more than two groups, analysis of variance was performed with post-hoc analysis (Fisher’s least squares difference test) for subgroup comparison. For the multivariable analysis, a linear regression analysis was performed to check the independence of the variables. All statistical analyses were performed using SPSS (version 25.0, Statistical Package for the Social Sciences, International Business Machines, Inc., Armonk, New York, USA). A two-sided *P*-value less than 0.05 was considered statistically significant.

## Results

### Baseline and genetic characteristics

The mean age of the 133 participants was 58 ± 13 years, and 35 (26%) of them were female. 34 (26%) had obstructive HCM, and 66 (50%) had apical pHCM. Of those with apical HCM, 43 (65%) participants had pure-type apical HCM. Based on ACMG guidelines [21], 41 (31%) participants had 43 pathogenic or likely pathogenic SMs (19 *MYBPC3*, 12 *MYH7*, 8 *TNNI3*, 2 *MYH6*, 1 *JPH2*, and 1 *TNNC1*). Two patients harbored more than one SMs (one had *MYBPC3* and *MYH7*; another had *MYBPC3* and *JPH2*). In total, 18 (14%) patients had a probably damaging mtDNA variant, and 11 (8%) had a pathogenic or likely pathogenic mitochondria-related nDNA variant. Six patients (5%) had both pathogenic or likely pathogenic SM and pathogenic MM. Of the 92 non-SM patients, 32 (35%) had any sarcomere VUS (Additional file 2: Table S1). The SM group included more women and had a lower prevalence of apical HCM and a higher prevalence of atrial fibrillation, as well as greater left atrial (LA) volume index, maximal LV thickness, and 5-year sudden cardiac death risk compared with non-SM group. Within non-SM group, patients with sarcomere VUS had higher echocardiographic Doppler E/e’ than others (Table 1). Age, sex, LA volume index, LV mass index, resting LV outflow tract gradient, percent LGE mass, LGE segment number, and global native and post-contrast T1 were significantly correlated with echocardiographic Doppler E/e’ (all *P* < 0.05). Among them, resting LV outflow tract gradient (β = 0.286; *P* < 0.001) and LGE segment number (β = 0.195; *P* = 0.037) were independently related to Doppler E/e’. The mitochondrial questionnaire score was not significantly different between the MM group and others. The age of normal control and positive control was 31 ± 2 years and 38 ± 19 years, respectively and their average 16-segmental ECV was 25.2 ± 1.6% and 29.3 ± 5.9% (average mid-ventricular ECV was 24.7 ± 2.4% and 28.4 ± 5.8%, respectively) was significantly lower than HCM patients (p < 0.001).

**Table 1:** Comparison of clinical and echo-Doppler findings between the sarcomere gene mutation group and the non-mutation group

	All HCM			Absence of pathogenic/likely pathogenic sarcomere gene mutation group		
	Presence of sarcomere gene mutation group (n = 41)	Absence of sarcomere gene mutation group (n = 92)	P	Sarcomere VUS (n = 32)	Absence of sarcomere VUS (n = 60)	P
Age, years	55.9 ± 13.5	59.6 ± 12.8	0.137	60.0 ± 12.5	59.4 ± 13.1	0.807
Women, n (%)	16 (39)	19 (21)	0.026	8 (25)	11 (18)	0.589
Hypertension, n (%)	281 (51)	39 (42)	0.345	15 (47)	24 (40)	0.658
Diabetes, n (%)	7 (17)	19 (21)	0.631	7 (22)	12 (20)	> 0.999
Persistent AF at echo, n (%)	9 (22)	7 (8)	0.019	3 (9)	4 (7)	0.691
Apical HCM, n (%)	13 (32)	53 (58)	0.006	14 (44)	39 (65)	0.076
Dynamic obstruction, n (%)	7 (17)	27 (29)	0.134	10 (31)	17 (28)	0.813
LA volume index, mL/m^2^	41.2 ± 17.6	32.4 ± 11.3	0.005	35.2 ± 12.8	31.0 ± 10.2	0.109
Echo Doppler E/e’	15.4 ± 7.3	14.6 ± 5.3	0.454	16.3 ± 5.6	13.7 ± 5.0	0.036
Maximal thickness, mm	19.9 ± 3.7	18.4 ± 3.6	0.032	18.5 ± 3.3	18.4 ± 3.8	0.911
5-year SCD risk (n = 83), %	2.8 ± 1.5	1.9 ± 1.8	0.029	2.4 ± 3.0	1.7 ± 0.6	0.214

*AF* atrial fibrillation, *HCM* hypertrophic cardiomyopathy, *E* early diastolic transmitral inflow velocity, *e’* early diastolic mitral annular velocity, *LA* left atrial, *SCD* sudden cardiac death, *VUS* variant uncertain significance

### Pathogenic sarcomere mutations on global and segmental LGE, ECV, and T2

**Fig. 1:**
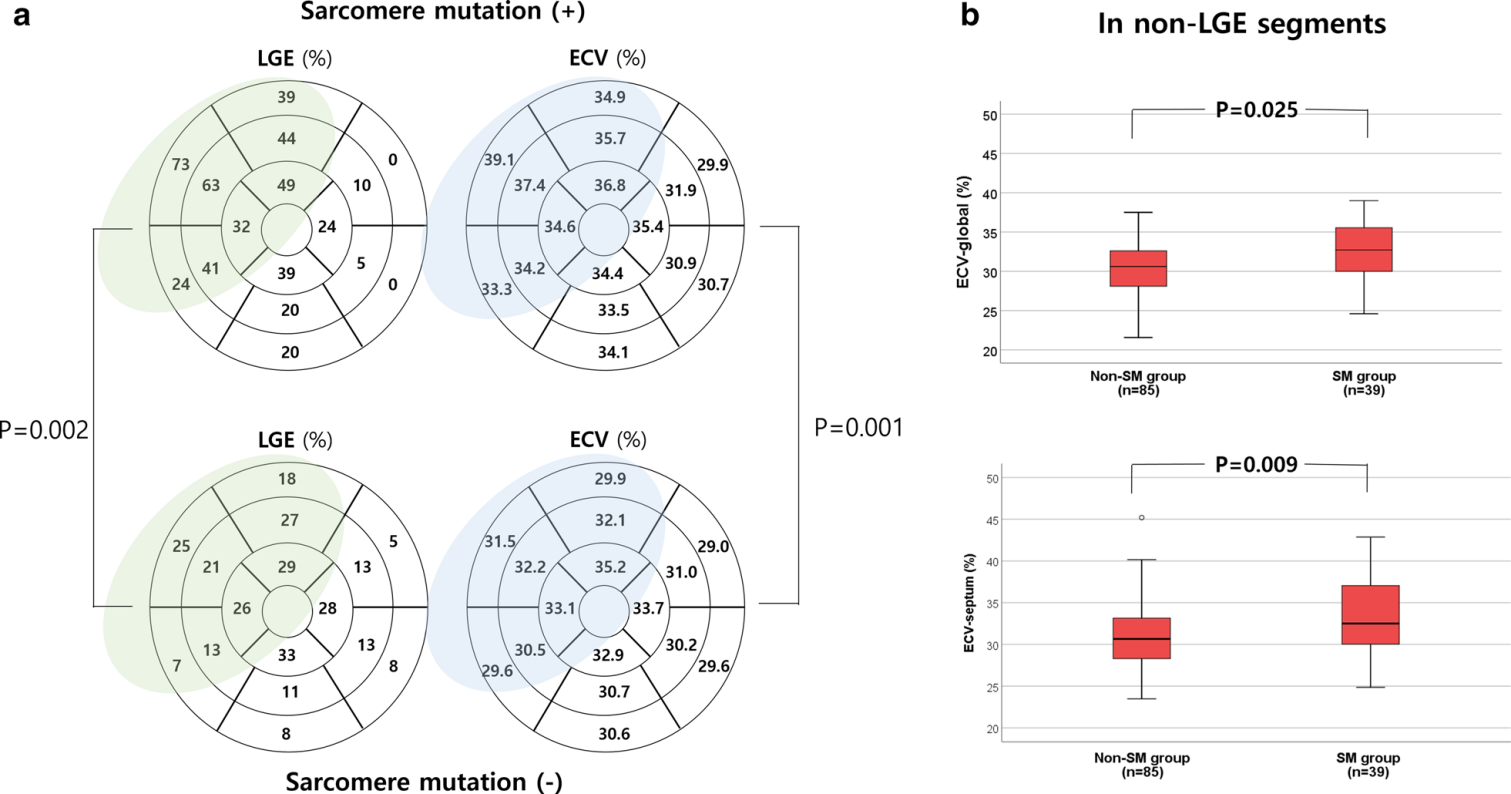
**a** Comparison of late gadolinium enhancement (LGE) involvement and extracellular volume fraction (ECV) between pathogenic or likely pathogenic sarcomere gene mutation (SM) group and non-SM group in all segments. **b** Comparison of global and septal ECV between SM group and non-SM group in segments without LGE

No significant difference in LV mass was found between the SM and the non-SM groups. However, the regional anteroseptal wall was significantly thicker in the SM group (13.2 ± 2.6 mm vs. 12.0 ± 3.4; *P* = 0.018), whereas the lateral wall was thicker in the non-SM group. Patients with pathogenic or likely pathogenic SMs had a higher prevalence of LGE (90% vs. 60%; *P* < 0.001) and more LGE-involved segments (4.9 ± 2.8 vs. 2.9 ± 3.5; *P* = 0.002) than patients without SM. The SM group had significantly higher global ECV than the non-SM group (34.2 ± 4.8% vs. 31.4 ± 4.3%; *P* = 0.001). In particular, the SM group had significantly higher ECV in septal segments (35.7 ± 6.7% vs. 31.4 ± 4.1%; *P* < 0.001) and anteroseptal segments (35.8 ± 6.8% vs. 31.8 ± 4.4%; *P* = 0.001) (Fig. 1). In addition, the SM group had shorter global and anteroseptal segment post-contrast T1 than the non-SM group. However, no difference in native T1 and T2 values were found between the SM and non-SM groups (Table 2). When analyzed in segments without LGE involvement, the SM group had significantly higher global (32.4 ± 3.8% vs. 30.7 ± 3.9%, *P* = 0.025), septal (33.3 ± 4.6% vs. 31.0 ± 3.9%; *P* = 0.006), anteroseptal, and inferoposterior ECV than the non-SM group (all *P* < 0.05; Fig. 1).

**Table 2:** Comparison of cardiovascular magnetic resonance imaging findings between the sarcomere gene mutation group and the non-mutation group

	Presence of sarcomere gene mutation group (n = 41)	Absence of sarcomere gene mutation group (n = 92)	*P*
LVEDV, mL	133.6 ± 28.6	135.7 ± 28.2	0.698
LVESV, mL	51.7 ± 23.1	46.9 ± 18.5	0.208
LV mass, g	153.7 ± 37.2	152.8 ± 48.0	0.913
LV mass index, g/m^2^	88.0 ± 21.1	84.7 ± 23.8	0.456
LV mass/volume ratio	1.19 ± 0.32	1.14 ± 0.32	0.469
LV ejection fraction, %	62.5 ± 9.4	65.7 ± 9.8	0.077
Segmental^*^ thickness, mm			
Thickness in anteroseptal segments	13.0 ± 2.9	11.7 ± 3.2	0.025
Thickness in septal segments	14.4 ± 3.4	12.5 ± 3.6	0.005
Thickness in inferoposterior segments	8.6 ± 2.1	8.9 ± 1.8	0.484
Thickness in lateral segments	7.6 ± 2.2	8.6 ± 2.1	0.020
Thickness in apical segments	10.5 ± 3.2	10.7 ± 3.9	0.771
*Late gadolinium enhancement (LGE)*			
Presence of LGE in LV, n (%)	37(90)	55(60)	< 0.001
Number of LGE segments in LV	4.9 ± 2.8	2.9 ± 3.5	0.002
% LGE amount of LV	10.6 ± 10.1	6.4 ± 9.3	0.040
LGE in anteroseptal segments, %	46.3 ± 25.7	20.7 ± 26.2	< 0.001
LGE in septal segments, %	47.8 ± 30.0	18.0 ± 26.2	< 0.001
LGE in inferoposterior segments, %	7.8 ± 14.8	13.5 ± 24.5	0.609
LGE in lateral segments, %	16.6 ± 20.4	14.4 ± 24.3	0.171
LGE in apical segments, %	36.0 ± 37.9	29.1 ± 37.5	0.331
*T2, ms*			
T2 average of 16 segments	56.1 ± 3.5	55.3 ± 3.0	0.177
T2 in anteroseptal segments	56.9 ± 3.7	55.3 ± 3.0	0.242
T2 in septal segments	57.9 ± 4.0	57.2 ± 3.7	0.360
T2 in inferoposterior segments	55.9 ± 3.7	54.8 ± 3.3	0.117
T2 in lateral segments	54.9 ± 4.0	54.0 ± 3.3	0.193
T2 in apical segments	57.1 ± 5.6	56.7 ± 4.3	0.677
*Native T1, ms*			
T1 average of 16 segments	1025 ± 47	1019 ± 49	0.512
T1 in anteroseptal segments	1023 ± 46	1017 ± 51	0.524
T1 in septal segments	10343 ± 44	1024 ± 49	0.301
T1 in inferoposterior segments	1045 ± 59	1032 ± 54	0.216
T1 in lateral segments	1014 ± 59	1013 ± 54	0.921
T1 in apical segments	1009 ± 66	1017 ± 58	0.469
*Post contrast T1, ms*			
T1 average of 16 segments	579 ± 59	607 ± 63	0.021
T1 in anteroseptal segments	569 ± 66	603 ± 64	0.008
T1 in septal segments	574 ± 66	608 ± 63	0.007
T1 in inferoposterior segments	595 ± 53	616 ± 63	0.065
T1 in lateral segments	590 ± 56	611 ± 65	0.085
*T1 in apical segments*	*564 ± 57*	588 ± 66	0.051
*Extracellular volume fraction (ECV), %*			
ECV average of 16 segments	34.2 ± 4.8	31.4 ± 4.3	0.001
ECV in anteroseptal segments	35.8 ± 6.8	31.8 ± 4.4	0.001
ECV in septal segments	35.7 ± 6.7	31.4 ± 4.1	< 0.001
ECV in inferoposterior segments	32.7 ± 4.0	30.8 ± 4.5	0.022
ECV in lateral segments	31.8 ± 3.9	30.7 ± 5.1	0.235
ECV in apical segments	35.3 ± 4.6	33.7 ± 5.5	0.118

*LV* left ventricular, *LVEDV* LV end-diastolic volume, *LVESV* LV end-systolic volume

### Effect of individual sarcomere, mitochondria-related mutations and VUS on myocardial fibrosis

**Fig. 2:**
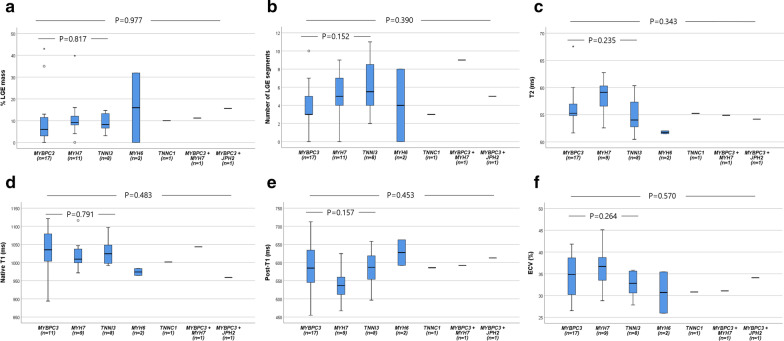
Comparisons of global percent LGE mass (**a**), LGE-involved segment number (**b**), T2 (**c**), native T1 (**d**), post contrast T1 (**e**), and ECV (**f**) among individual sarcomere variants

**Fig. 3:**
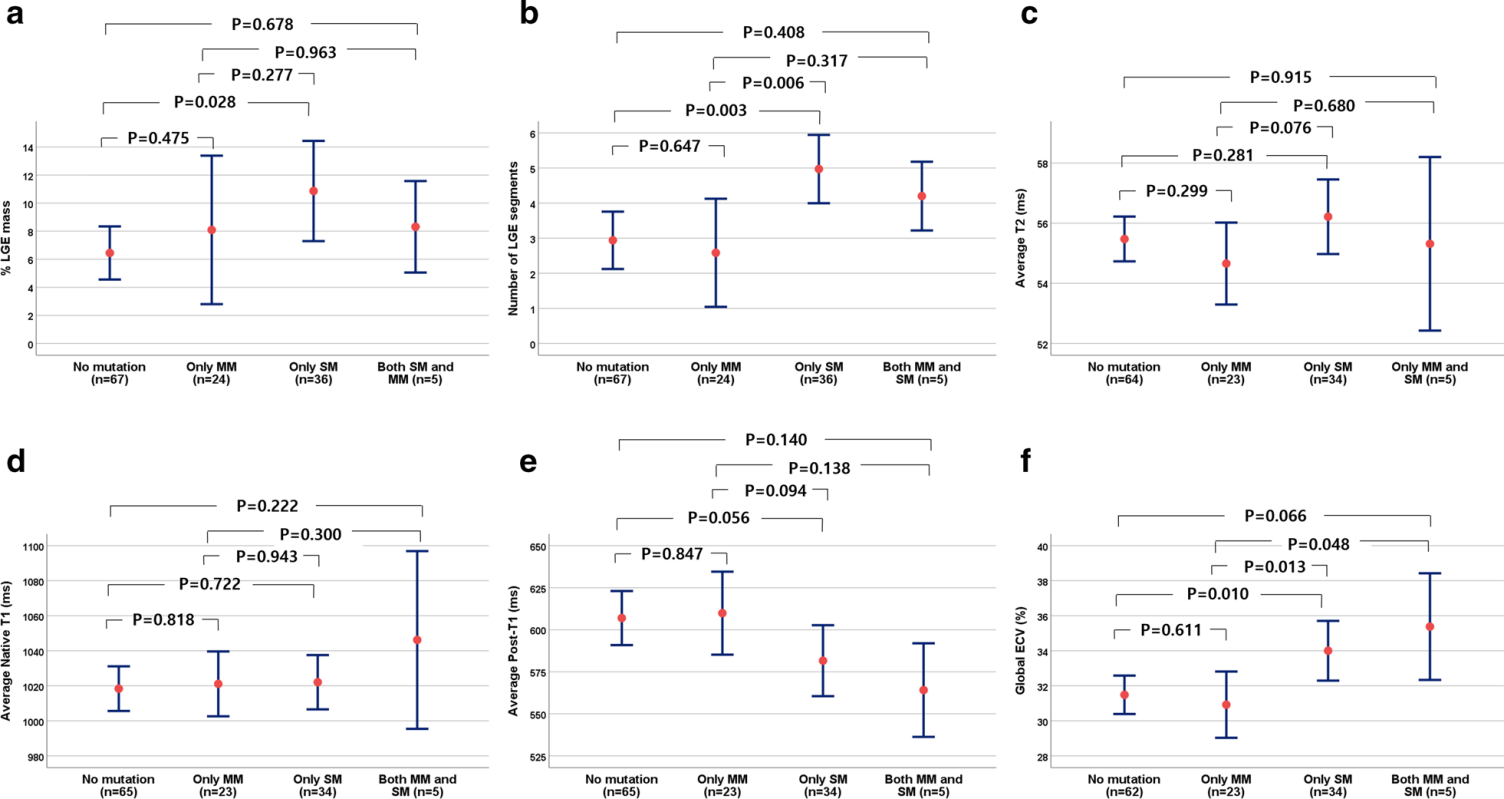
Comparisons of global %LGE (**a**), LGE-involved segment number (**b**), T2 (**c**), native T1 (**d**), post contrast T1 (**e**), and ECV (**f**) between sarcomere and mitochondria-related variant groups. *SM* sarcomere mutation group, *MM* mitochondria-related mutation group

Although patients with pathogenic or likely pathogenic *MYH7* mutations tended to have higher %LGE and ECVs than those with *MYBPC3* or other SMs, this difference did not reach significance (Fig. 2). Detailed genetic alterations of detected sarcomere-associated genes, mitochondria-related nDNAs and damaging mtDNA variants are shown in Additional file 2: Tables S2 and S3. Known pathogenic mtDNA variants were detected from only four patients and were not present with extracardiac features of mitochondrial disease such as diabetes, deafness and etc. When classified into non-mutation (n = 67), only MM (n = 24), only-SM (n = 36), and both SM and MM (n = 5) groups (one patient was missed due to non-analysis of mtDNA), only-SM group had higher amount of LGE and ECV compared to non-mutation group and only-MM group (all p < 0.05). The MM-only group was not significantly different from the non-mutation group (Table 3; Fig. 3; Additional file 2). For the non-SM group, patients with sarcomere VUS demonstrated a trend for a higher %LGE and ECV (Additional file 2: Table S4).

**Table 3:** Comparison of cardiac magnetic resonance imaging findings between the sarcomere gene and mitochondria-related gene mutation groups

	No pathogenic or likely pathogenic variant group (n = 67)	Only mitochondria-related nDNA or mtDNA variant group^a^ (n = 24)	Only sarcomere gene variant group (n = 36)	Sarcomere and mitochondria-related gene variant group^a^ (n = 5)	*P*
*Late gadolinium enhancement (LGE)*					
Presence of LGE in left ventricle	42 (61)	12 (50)	32 (89)	5 nn	0.003
Number of LGE segments in left ventricle	2.9 ± 3.3	2.6 ± 3.8	5.0 ± 2.9^**,††^	4.2 ± 1.1	0.012
% LGE amount of left ventricle	6.4 ± 7.8	8.1 ± 13.0	10.9 ± 10.7^*^	8.3 ± 3.6	0.184
LGE in anteroseptal segments, %	20.9 ± 24.9	17.7 ± 28.1	47.2 ± 27.2^**,††^	40.0 ± 5.6^†^	< 0.001
LGE in septal segments, %	18.2 ± 24.8	15.0 ± 27.8	48.3 ± 31.5^**,††^	44.0 ± 16.7^*,††^	< 0.001
LGE in inferoposterior segments, %	12.8 ± 23.8	15.0 ± 27.2	7.8 ± 14.6	8.0 ± 17.9	0.826
LGE in lateral segments, %	15.2 ± 25.4	11.7 ± 22.0	17.2 ± 20.9	12.0 ± 17.9	0.580
LGE in apical segments, %	28.4 ± 37.7	28.1 ± 35.6	37.5 ± 39.4	25.0 ± 25.0	0.643
*Extracellular volume fraction (ECV), %*					
ECV average of 16 segments	31.5 ± 4.3	30.9 ± 4.5	34.0 ± 5.0^*,†^	35.4 ± 3.4^†^	0.013
ECV in anteroseptal segments	31.9 ± 4.2	31.4 ± 5.0	35.6 ± 7.0^**,††^	36.9 ± 5.2^*,†^	0.002
ECV in septal segments	31.6 ± 3.9	30.7 ± 4.8	35.6 ± 6.9^**,††^	36.8 ± 5.8^*,†^	< 0.001
ECV in inferoposterior segments	31.0 ± 4.6	30.2 ± 4.0	32.4 ± 4.1	34.8 ± 2.7^†^	0.065
ECV in lateral segments	30.9 ± 5.2	30.0 ± 4.8	31.7 ± 4.1	32.4 ± 1.8	0.544
ECV in apical segments	33.7 ± 5.2	33.7 ± 6.4	35.3 ± 4.8	35.5 ± 4.3	0.500

One patient was missed due to non-analysis of mtDNA

^a^Pathogenic or likely pathogenic mitochondria-related nDNA mutations or damaging mtDNA variants

^*^P < 0.05

**P < 0.01 versus no pathogenic or likely pathogenic variant group

^†^*P* < 0.05

^††^*P* < 0.01 versus mitochondria-related variant group

## Discussion

This study confirmed that pathogenic or likely pathogenic SMs are significantly related to higher amounts of LV replacement fibrosis and ECV, especially in the septal area. This finding supports that worse outcomes in patients with HCM are related to SMs. Even in segments without LGE, ECV was significantly higher in patients with pathogenic or likely pathogenic SMs. Within non-SM group, patients with any sarcomere VUS had higher Doppler E/e’ and tendency of higher fibrosis. However, MMs were not related to increased myocardial fibrosis.

### Sarcomere mutation and myocardial fibrosis

In a previous study by Ellims, although patients with SMs had higher LGE amounts, they had longer post-T1 than those without SMs, especially in non-LGE segments [6]. This result differed from that of other studies [2, 7]. In our study, patients with SMs had higher ECV than those without SM—not only globally and regionally in the septum but also in lateral and inferolateral segments where LGE involvement is rare. These results suggest higher amounts of diffuse interstitial fibrosis in patients with SM, which is consistent with other studies [2, 7, 8]. Regarding SM related worse phenotypes, the NHLBI HCM registry results reported that SM was more likely to have reverse septal curvature morphology, LGE, and no significant resting LV outflow tract obstruction, while those that were SM negative were more likely to have isolated basal septal hypertrophy, less LGE, and more LV outflow tract obstruction [7]. Our study adds a new evidence of higher prevalence of apical type HCM, known as benign phenotype in SM negative group. Our study results are compatible with the previous studies that SM positive group has higher amount of replacement fibrosis and interstitial fibrosis[7], even in non-LGE segments, which supports worse prognosis, especially for significant arrhythmic events due to potential substrates for reentry circuit due to tissue heterogeneity [8, 9]. In addition, we found that Doppler E/e’ was significantly correlated with LGE (both %LGE and the number of LGE-involved segments), as well as regional ECV (septal and anteroseptal segments). A previous study revealed that a sarcomere gene (*MYH7*) mutation, induces profibrotic change and fibrosis through activation of TGF-β signaling in non-myocyte cells of mice myocardium [22]. It suggested that myocardial fibrosis would be a primary phenotype of sarcomere mutations, which is also supported by our study results, in terms of replacement fibrosis (LGE) and interstitial fibrosis (ECV). Interestingly, within non-SM group, patients with VUS had higher E/e’ and tendency of higher LGE amount and ECV, which supports a previous study results of worse prognosis. But it needs further investigation with large number of population [9]. However, the T2 value was not significantly different among genetic-based subgroups, suggesting that occult myocardial inflammation is not a primary phenotype in HCM. One also has to recognize that absence of known SM does not mean that there is no mutation, it only means the mutation in the patient has not been broadly identified or recognized as a likely disease causing mutation.

### Mitochondria-related mutations and myocardial fibrosis

Basic science studies have shown that MMs induce pro-arrhythmic effects and myocardial dysfunction, especially in diastolic function [23]. However, no studies have observed the effect of MM on myocardial fibrosis. According to our study results, some patients had pathogenic MMs in patients without SM or with SM. However, patients with MM in the non-SM group exhibited no significant differences in clinical parameters, degree of replacement fibrosis, or diffuse interstitial fibrosis and inflammation (as reflected by LGE, ECV, and T2 values) from those without any mutations.

Regarding whether MMs have additive effects with SMs on myocardial fibrosis, patients with both sarcomere and mitochondria-related mutations tended to have higher ECVs. However, no significant difference was seen, possibly due to the subgroup’s small size (5% of the entire sample). This result suggests that a MM has benign phenotypic characteristics or does not contribute to phenotypic expressions because of recessive heritance and heteroplasmy of mtDNA mutations [10]. In addition, it suggests that SM, not MM, contributes to the primary phenotypes of HCM, myocardial scarring and diffuse fibrosis. Regarding the effects of MMs on phenotypic changes in HCM, consideration for assessing nDNA and mtDNA mutation load is needed. As shown in a previous study related to mitochondrial cardiomyopathy, interstitial and replacement fibrosis is rarely seen [10], so the contribution of MMs to myocardial fibrosis would be minimal. Although, in our study MM was not significantly related to myocardial fibrosis, development of potential or hidden endophenotype should be closely followed [11, 24].

### Regional extracellular space expansion in segments without LGE

Even in segments without LGE, ECV was significantly higher in patients with SMs. This finding suggests that before the development of scarring or replacement fibrosis, diffuse interstitial fibrosis develops in this HCM group and supports that interstitial fibrosis is a primary phenotype in SM-positive HCM. Therefore, regional ECV assessment and serial LGE imaging follow-up may be suitable for risk stratification in HCM patients. The potential mechanism of action may be impaired myocardial flow reserve due to small intramuscular coronary artery constriction, but our study did not include the stress myocardial perfusion protocol in CMR. Thus, future studies on this topic are warranted.

## Limitations

Our study had several limitations. Due to our inclusion of a large portion of patients with apical HCM, the prevalence of pathogenic SM was low. However, within patients without apical HCM, the pathogenic SM rate was consistent with other studies. Likewise, the comparison between the two major SMs, *MYBPC3* and *MYH7,* was not sufficient due to the small sample size. However, similar to recent results from a multicenter registry [9], we found trend of higher fibrosis in patients with the *MYH7* mutation than in those with the *MYBPC3* mutation. The ECV value of HCM was slightly higher than other studies, it may be due to algorithm of analysis program. However, in this study, all of the patients underwent native T1 mapping using the same scanner, same protocol, and same analysis program, meaning the impact of genetic mutation on ECV would not be changed. Finally, only a small number of healthy controls were recruited due to significant social limitations at our local institution and the need for gadolinium contrast phlebotomy for hematocrit. While not ideal, from a practical standpoint, young people with idiopathic ventricular tachycardia and aborted sudden cardiac death became part of the de facto control group in this study.

## Conclusion

SMs are significantly related to increased myocardial fibrosis, even in segments without LGE supporting the findings of a worse prognosis in HCM patients with SM. Our findings also support that myocardial fibrosis is a primary phenotype in patients with SMs. Within non-SM group, patients with any VUS had higher Doppler E/e’ and tendency of higher LGE amount and ECV, which supports a previous study results of worse prognosis in patients with VUS. Although some patients with HCM had pathogenic MMs, they did not exhibit increased myocardial fibrosis.

## Supplementary Information

**Additional file 1: Method S1.** DNA preparation, library construction and sequencing of the HCM gene panel and mtDNA. **Method S2.** Detail questions for assessment of systemic involvement of mitochondrial dysfunction. **Method S3.** Echocardiographic analysis.

**Additional file 2: Table S1.** Nonsynonymous variants in the 33 sarcomere associated genes classified according to the refined American College of Medical Genetics and Genomics (ACMG) standards and guidelines for inherited cardiac conditions. **Table S2.** Likely pathogenic or Pathogenic variants in the 6 non-sarcomere genes and the 44 mitochondria-related nuclear gene. **Table S3.** Mitochondrial DNA mutations upto probably damaging. **Table S4.** Comparisons of cardiac magnetic resonance imaging findings between the patients with and without any sarcomere variants of uncertain significance within patients without any pathogenic or likely pathogenic sarcomere gene variants

## Data Availability

The datasets used and/or analyzed during the current study are available from the corresponding author on reasonable request.

## Abbreviations

ACMG: American College of Medical Genetics and Genomics
AHA: American Heart Association
bSSFP: Balanced steady state free precession
CMR: Cardiovascular magnetic resonance
ECV: Extracellular volume fraction
HCM: Hypertrophic cardiomyopathy
LA: Left atrium
LGE: Late gadolinium enhancement
LV: Left ventricle/left ventricular
LVEDV: Left ventricular end-diastolic volume
LVESV: Left ventricular end-systolic volume
MM: Mitochondria-related gene mutations
MOLLI: Modified Look-Locker inversion recovery
mtDNA: Mitochondrial DNA
nDNA: Nuclear DNA
PSIR: Phase sensitive inversion recovery
SM: Sarcomere gene mutation
VUS: Variants of unknown sequence
