# Supplementary material for: Effect of sarcomere and mitochondria-related mutations on myocardial fibrosis in patients with hypertrophic cardiomyopathy
Source: J Cardiovasc Magn Reson. 2021 Mar 4;23:18. doi: 10.1186/s12968-021-00718-3 (PMC7931545; doi:10.1186/s12968-021-00718-3)
Supplement: Supplementary file 1 — Additional file 1: Method S1. DNA preparation, library construction and sequencing of the HCM gene panel and mtDNA. Method S2. Detail questions for assessment of systemic involvement of mitochondrial dysfunction. Method S3. Echocardiographic analysis. [file 12968_2021_718_MOESM1_ESM.docx]

**Method S1. DNA preparation, library construction and sequencing of the HCM gene panel and mtDNA**

Genomic DNA was extracted from EDTA-treated whole blood samples by using a QIAamp DNA Blood Mini kit (Qiagen, Hilden, Germany) on a QIAcube automatic nucleic acid extrac­tion instrument (Qiagen) according to the manufacturer’s in­structions. The DNA samples were used to analyze mtDNA and the HCM gene panel (nDNA). For targeted sequencing of the HCM gene panel, DNA fragments were enriched by solution-based hybridization capture and sequenced on an Illumina Hiseq2500 platform (Illumina, San Diego, CA, USA) with the 2 × 150 base pair paired-end read module. Library preparation, hybridization, capture procedure, and sequencing were performed by Celemics according to the protocols recommended by the Celemics User Manual Ver 2.1 (<http://www.celemics.com/home/>).

The complete mtDNA was amplified by using four overlapping pairs of primers.(1) Library preparations were performed following the manufacturer’s instructions (Ion XpressPlus Fragment Library Kit; Thermo Fisher Scientific, Waltham, MA, USA) for 400 single-end reads. Sequencing was conducted on the 318 chip using Ion PGM Hi-Q Sequencing Kit on the Ion Torrent Personal Genome Machine (Thermo Fisher Scientific). Data analysis of mtDNA and the HCM gene panel were performed, as previously described. Visual inspection of the mapped data was performed using Integrated Genomics Viewer 2.3 software (IGV; Broad Institute, Cambridge, MA, USA).

**Method S2. Detail questions for assessment of systemic involvement of mitochondrial dysfunction**

For neurologic involvement, 10 questions addressed headache, previous history of stroke, history of epilepsy or seizure, motor weakness of extremities, sensory changes, presence of diplopia, gait disturbance, dysphasia, and hearing difficulties. For gastrointestinal involvement, 3 questions addressed constipation, diarrhea, and dysphagia. For endocrinal abnormalities, 3 questions addressed history of diabetes, thyroid disease, and infertility. For ophthalmologic abnormalities, 3 questions addressed visual disturbance, blurred vision, and ptosis. The numbers of “yes” responses were summed, and the score was used for analysis. (2)

**Method S3. Echocardiographic analysis**

Echocardiography analysis was performed with a commercially available machine. Comprehensive echo-Doppler evaluation was performed according to current American Society of Echocardiography guidelines.(3) Simpson’s method was used to calculate LV ejection fraction. LV wall thickness was measured in all cross-sectional planes. The maximal thickness of LV was defined as the largest dimension evident at any site within the LV chamber. Posterior wall thickness was measured at the parasternal long axis view or short axis view. A continuous wave Doppler was used to measure peak velocity across the LV outflow tract (LVOT), and the pressure gradient (PG) was calculated by using the Bernoulli equation, 4 × (peak velocity across the LVOT)^2^. This value was measured at rest and during Valsalva maneuver. LVOT obstruction was defined as a systolic PG of 30 mmHg or higher across the LVOT. PG was also measured at the mid-level of the LV cavity. To improve the LV border definition, contrast echocardiography was performed in patients with poorly defined LV borders. We classified the obstructive HCM according to the presence of LVOT or mid-LV obstruction, either resting or dynamic condition.

**References**

1. Wang HW, Jia X, Ji Y, Kong QP, Zhang Q, Yao YG, et al. Strikingly different penetrance of LHON in two Chinese families with primary mutation G11778A is independent of mtDNA haplogroup background and secondary mutation G13708A. *Mutation research*. 2008 Aug 25;**643**(1-2):48-53. PubMed PMID: 18619472. Epub 2008/07/16. eng.

2. Mattman A, Sirrs S, Mezei MM, Salvarinova-Zivkovic R, Alfadhel M, Lillquist Y. Mitochondrial disease clinical manifestations: An overview. *British Columbia Medical Journal*. 2011;**53**(4):183-7. PubMed PMID: 63614776.

3. Lang RM, Bierig M, Devereux RB, Flachskampf FA, Foster E, Pellikka PA, et al. Recommendations for chamber quantification: a report from the American Society of Echocardiography's Guidelines and Standards Committee and the Chamber Quantification Writing Group, developed in conjunction with the European Association of Echocardiography, a branch of the European Society of Cardiology. *Journal of the American Society of Echocardiography : official publication of the American Society of Echocardiography*. 2005 Dec;**18**(12):1440-63. PubMed PMID: 16376782.
