# Supplementary material for: Effect of sarcomere and mitochondria-related mutations on myocardial fibrosis in patients with hypertrophic cardiomyopathy
Source: J Cardiovasc Magn Reson. 2021 Mar 4;23:18. doi: 10.1186/s12968-021-00718-3 (PMC7931545; doi:10.1186/s12968-021-00718-3)
Supplement: Supplementary file 2 — Additional file 2: Table S1. Nonsynonymous variants in the 33 sarcomere associated genes classified according to the refined American College of Medical Genetics and Genomics (ACMG) standards and guidelines for inherited cardiac conditions. Table S2. Likely pathogenic or Pathogenic variants in the 6 non-sarcomere genes and the 44 mitochondria-related nuclear gene. Table S3. Mitochondrial DNA mutations upto probably damaging. Table S4. Comparisons of cardiac magnetic resonance imaging findings between the patients with and without any sarcomere variants of uncertain significance within patients without any pathogenic or likely pathogenic sarcomere gene variants [file 12968_2021_718_MOESM2_ESM.docx]

**Table S1.** Nonsynonymous variants in the 33 sarcomere associated genes classified according to the refined American College of Medical Genetics and Genomics (ACMG) standards and guidelines for inherited cardiac conditions.

| Case | GENE | Reference sequence | DNA Change | Amino Acid Change | Zygosity | ACMG classification |
| --- | --- | --- | --- | --- | --- | --- |
| YMC-11 | *MYH6* | NM_002471.3 | c.4769G>A | p.Arg1590His | Heterozygous | VUS |
| YMC-15 | *TNNI3* | NM_000363.4 | c.434G>A | p.Arg145Gln | Heterozygous | Pathogenic |
| YMC-16 | *MYOM1* | NM_003803.3 | c.1351dupT | p.Ser451Phefs*27 | Heterozygous | VUS |
|  | *MYH7* | NM_000257.3 | c.3852T>C | p.Asn1284= | Heterozygous | VUS |
|  | *OBSCN* | NM_052843.3 | c.3662G>A | p.Arg1221Gln | Heterozygous | VUS |
| YMC-17 | *MYBPC3* | NM_000256.3 | c.2512G>T | p.Glu838* | Heterozygous | Pathogenic |
| YMC-20 | *MYH7* | NM_000257.3 | c.4130C>T | p.Thr1377Met | Heterozygous | Likely pathogenic |
|  | *OBSCN* | NM_052843.3 | c.11225C>T | p.Pro3742Leu | Heterozygous | VUS |
| YMC-21 | *MYOM1* | NM_003803.3 | c.970G>C | p.G324R | Heterozygous | VUS |
| YMC-22 | *MYH7* | NM_000257.3 | c.5704G>C | p.Glu1902Gln | Heterozygous | VUS |
|  | *OBSCN* | NM_052843.3 | c.16706C>T | p.Thr5569Ile | Heterozygous | VUS |
| YMC-25 | *MYBPC3* | NM_000256.3 | c.1000G>A | p.E334K | Heterozygous | Likely pathogenic |
| YMC-27 | *TNNI3* | NM_000363.4 | c.434G>A | p.Arg145Gln | Heterozygous | Pathogenic |
| YMC-32 | *MYBPC3* | NM_000256.3 | c.86delT | p.Phe29Serfs*10 | Heterozygous | Pathogenic |
| YMC-34 | *BAG3* | NM_004281.3 | c.652C>T | p.Arg218Trp | Heterozygous | VUS |
| YMC-40 | *MYH7* | NM_000257.3 | c.4130C>T | p.Thr1377Met | Heterozygous | Likely pathogenic |
| YMC-41 | *MYBPC3* | NM_000256.3 | c.2833_2834del | p.Arg945Glyfs | Heterozygous | Pathogenic |
| YMC-43 | *MYBPC3* | NM_000256.3 | c.1505G>A | p.Arg502Gln | Heterozygous | Pathogenic |
|  | *JPH2* | NM_020433.4 | c.1037_1047del | p.Leu346Hisfs*50 | Heterozygous | Pathogenic |
| YMC-48 | *MYBPC3* | NM_000256.3 | c.2504G>T | p.Arg835Leu | Heterozygous | VUS |
| YMC-51 | *MYBPC3* | NM_000256.3 | c.2067+1G>A | (-) | Heterozygous | Pathogenic |
| YMC-52 | *MYH7* | NM_000257.3 | c.599C>T | p.Ala200Val | Heterozygous | Likely pathogenic |
| YMC-53 | *ACTC1* | NM_005159.4 | c.931A>G | p.Ile311Val | Heterozygous | VUS |
|  | *MYBPC3* | NM_000256.3 | c.3316G>A | p.Asp1106Asn | Heterozygous | VUS |
| YMC-54 | *TNNI3* | NM_000363.4 | c.485G>C | p.Arg162Pro | Heterozygous | Likely pathogenic |
|  | *ACTN2* | NM_001103.2 | c.1343A>C | p.Glu448Ala | Heterozygous | VUS |
| YMC-59 | *MYH6* | NM_002471.3 | c.2423A>G | p.Glu808Gly | Heterozygous | VUS |
|  | *MYH6* | NM_002471.3 | c.4231G>A | p.Ala1411Thr | Heterozygous | VUS |
| YMC-67 | *OBSCN* | NM_052843.3 | c.17284G>A | p.Asp5762Asn | Heterozygous | VUS |
| YMC-68 | *MYBPC3* | NM_000256.3 | c.3072C>A | p.Ser1024Arg | Heterozygous | VUS |
| YMC-70 | *MYH6* | NM_002471.3 | c.1663G>A | p.Asp555Asn | Heterozygous | VUS |
| YMC-78 | *MYH7* | NM_000257.3 | c.4130C>T | p.Thr1377Met | Heterozygous | Likely pathogenic |
| YMC-79 | *TNNC1* | NM_003280.2 | c.23C>T | p.Ala8Val | Heterozygous | Likely pathogenic |
| YMC-80 | *MYH7* | NM_000257.3 | c.2606G>C | p.Arg869Pro | Heterozygous | Likely pathogenic |
| YMC-81 | *MYO6* | NM_004999.3 | c.1532A>G | p.Asn511Ser | Heterozygous | VUS |
|  | *MYOM1* | NM_003803.3 | c.637C>T | p.Gln213* | Heterozygous | VUS |
|  | *NEXN* | NM_144573.3 | c.767G>A | p.Arg256Gln | Heterozygous | VUS |
| YMC-85 | *MYBPC3* | NM_000256.3 | c.2067+1G>A | (-) | Heterozygous | Pathogenic |
| YMC-86 | *MYBPC3* | NM_000256.3 | c.3098G>A | p.Arg1033Gln | Heterozygous | VUS |
| YMC-87 | *VCL* | NM_014000.2 | c.1390A>G | p.Lys464Glu | Heterozygous | VUS |
| YMC-88 | *MYBPC3* | NM_000256.3 | c.3490+1G>A | (-) | Heterozygous | Pathogenic |
| YMC-90 | *MYBPC3* | NM_000256.3 | c.2833_2834del | p.Arg945Glyfs | Heterozygous | Pathogenic |
|  | *MYBPC3* | NM_000256.3 | c.2641G>A | p.Val881Ile | Heterozygous | VUS |
| YMC-92 | *MYH7* | NM_000257.3 | c.3064A>G | p.Lys1022Glu | Heterozygous | VUS |
|  | *OBSCN* | NM_052843.3 | c.6041C>A | p.Pro2014Gln | Heterozygous | VUS |
| YMC-93 | *MYH7* | NM_000257.3 | c.4130C>T | p.Thr1377Met | Heterozygous | Likely pathogenic |
|  | *OBSCN* | NM_052843.3 | c.11225C>T | p.Pro3742Leu | Heterozygous | VUS |
| YMC-95 | *TNNI3* | NM_000363.4 | c.434G>A | p.Arg145Gln | Heterozygous | Pathogenic |
|  | *TNNC1* | NM_003280.2 | c.304C>T | p.R102C | Heterozygous | VUS |
| YMC-100 | *MYH7* | NM_000257.3 | c.1426C>T | p.Leu476Phe | Heterozygous | VUS |
| YMC-101 | *OBSCN* | NM_052843.3 | c.12676G>T | p.Asp4226Tyr | Heterozygous | VUS |
| YMC-104 | *MYBPC3* | NM_000256.3 | c.3763delG | p.Ala1255Profs*76 | Heterozygous | Likely pathogenic |
| YMC-110 | *MYH6* | NM_002471.3 | c.679dupG | p.Ala227Glyfs*24 | Heterozygous | Likely pathogenic |
| YMC-111 | *MYBPC3* | NM_000256.3 | c.643C>T | p.Arg215Cys | Heterozygous | VUS |
| YMC-114 | *MYBPC3* | NM_000256.3 | c.1000G>A | p.E334K | Heterozygous | Likely pathogenic |
|  | *OBSCN* | NM_052843.3 | c.15586G>A | p.Val5196Ile | Heterozygous | VUS |
|  | *MYH7* | NM_000257.3 | c.1324C>T | p.Arg442Cys | Heterozygous | Likely pathogenic |
| YMC-117 | *TNNI3* | NM_000363.4 | c.434G>A | p.Arg145Gln | Heterozygous | Pathogenic |
| YMC-121 | *MYBPC3* | NM_000256.3 | c.1000G>A | p.E334K | Homozygous | Likely pathogenic |
|  | *MYOM1* | NM_003803.3 | c.970G>C | p.G324R | Heterozygous | VUS |
| YMC-122 | *TCAP* | NM_003673.3 | c.425C>G | p. Pro142Arg | Heterozygous | VUS |
| YMC-123 | *TNNI3* | NM_000363.4 | c.434G>A | p.Arg145Gln | Heterozygous | Pathogenic |
|  | *MYBPC3* | NM_000256.3 | c.3137C>T | p.Thr1046Met | Heterozygous | VUS |
| YMC-126 | *MYBPC3* | NM_000256.3 | c.1000G>A | p.E334K | Heterozygous | Likely pathogenic |
| YMC-127 | *MYBPC3* | NM_000256.3 | c.1000G>A | p.E334K | Heterozygous | Likely pathogenic |
|  | *MYPN* | NM_001256267.1 | c.3299C>T | p.Pro1100Leu | Heterozygous | VUS |
|  | *OBSCN* | NM_052843.3 | c.17555G>A | p.Arg5852Gln | Heterozygous | VUS |
| YMC-134 | *MYBPC3* | NM_000256.3 | c.2067+1G>A | (-) | Heterozygous | Pathogenic |
| YMC-135 | *TTN* | NM_001267550.1 | c.74840G>A | p.Arg24947His | Heterozygous | VUS |
| YMC-136 | *MYBPC3* | NM_000256.3 | c.2459G>A | p.Arg820Gln | Heterozygous | Likely pathogenic |
|  | *MYO6* | NM_004999.3 | c.3377C>A | p.Thr1126Lys | Heterozygous | VUS |
| YMC-143 | *MYH7* | NM_000257.3 | c.2981A>G | p.Lys994Arg | Heterozygous | VUS |
| YMC-144 | *MYBPC3* | NM_000256.3 | c.1090+1G>A | (-) | Heterozygous | Pathogenic |
| YMC-148 | *MYH6* | NM_002471.3 | c.2384G>A | p.Arg795Gln | Heterozygous | Likely pathogenic |
| YMC-150 | *TNNI3* | NM_000363.4 | c.434G>A | p.Arg145Gln | Heterozygous | Pathogenic |
| YMC-151 | *MYBPC3* | NM_000256.3 | c.2067+1G>A | (-) | Heterozygous | Pathogenic |
|  | *RYR2* | NM_001035.2 | c.5717T>C | p.Met1906Thr | Heterozygous | VUS |
| YMC-153 | *TTN* | NM_001267550.1 | c.42284C>T | p.Ser14095Phe | Heterozygous | VUS |
| YMC-154 | *MYH7* | NM_000257.3 | c.1988G>A | p.Arg663His | Heterozygous | Likely pathogenic |
| YMC-159 | *MYH7* | NM_000257.3 | c.1426C>T | p.Leu476Phe | Heterozygous | Likely pathogenic |
| YMC-161 | *OBSCN* | NM_052843.3 | c.6041C>A | p.Pro2014Gln | Heterozygous | VUS |
| YMC-173 | *RYR2* | NM_001035.2 | c.7076G>A | p.Arg2359Gln | Heterozygous | VUS |
| YMC-174 | *MYBPC3* | NM_000256.3 | c.1000G>A | p.Glu334Lys | Heterozygous | Likely pathogenic |
|  | *TPM1* | NM_001018005.1 | c.376G>A | p.Gly126Ser | Heterozygous | VUS |
| YMC-175 | *MYH7* | NM_000257.3 | c.746G>A | p.Arg249Gln | Heterozygous | Likely pathogenic |
|  | *MYH7* | NM_000257.3 | c.746G>A | p.Arg249Gln | Heterozygous | VUS |
| YMC-179 | *MYPN* | NM_032578.3 | c.3205C>T | p.Arg1069Cys | Heterozygous | VUS |
| YMC-180 | *TNNI3* | NM_000363.4 | c.434G>A | p.Arg145Gln | Heterozygous | Pathogenic |
|  | *ACTN2* | NM_001103.3 | c.1040C>T | p.Thr347Met | Heterozygous | VUS |
| YMC-183 | *VCL* | NM_014000.2 | c.379G>A | p.Asp127Asn | Heterozygous | VUS |
| YMC-194 | *FHL1* | NM_001449.4 | c.828T>G | p.Cys276Trp | Heterozygous | VUS |
| YMC-197 | *RYR2* | NM_001035.2 | c.5255G>A | p.Gly1752Asp | Heterozygous | VUS |
|  | *OBSCN* | NM_052843.3 | c.6041C>A | p.Pro2014Gln | Heterozygous | VUS |
| YMC-198 | *MYH7* | NM_000257.3 | c.4066G>A | p.Glu1356Lys | Heterozygous | Likely pathogenic |
| YMC-201 | *ANKRD1* | NM_014391.2 | c.545G>A | p.Arg182His | Heterozygous | VUS |
| YMC-202 | *MYOM1* | NM_003803.3 | c.4672T>C | p.Phe1558Leu | Heterozygous | VUS |
| YMC-207 | *MYH7* | NM_000257.3 | c.1615A>G | p.Met539Val | Heterozygous | Likely pathogenic |
| YMC-208 | *MYH6* | NM_002471.3 | c.4207G>A | p.Glu1403Lys | Heterozygous | VUS |
| YMC-208 | *TTN* | NM_001267550.1 | c.60620A>T | p.Tyr20207Phe | Heterozygous | VUS |
| YMC-212 | *RYR2* | NM_001035.2 | c.3766C>A | p.Pro1256Thr | Heterozygous | VUS |
|  | *TTN* | NM_001267550.1 | c.27856G>T | p.Val9286Phe | Heterozygous | VUS |

Abbreviations: ACMG, American College of Medical Genetics and Genomics; NM number, National Center for Biotechnology Information (NCBI) reference sequence; VUS, variant of uncertain significance;

**Table S2.** Likely pathogenic or Pathogenic variants in the 6 non-sarcomere genes and the 44 mitochondria-related nuclear genes

| Case | GENE | Reference sequence | DNA Change | Amino Acid Change | Align GVGD^a^ | SIFT^b^ | Mutation Taster^b^ | Clinvar accession | Clinvar phenotype | HGMD accession | HGMD phenotype | HGMD variant class | MAF (ExAC) | MAF (KRGDB) | dbSNP |
| --- | --- | --- | --- | --- | --- | --- | --- | --- | --- | --- | --- | --- | --- | --- | --- |
| YMC-12 | *AARS2* | NM_020745.3 | c.452T>C | p.Met151Thr | Class C0 (GV: 268.85 - GD: 38.84) | Tolerated (score: 0.07) | Disease causing (p-value: 1) | (-) | (-) | CM173156 | Ovarioleukodystrophy | DM | (-) | (-) | (-) |
|  | *ACADVL* | NM_000018.3 | c.865G>A | p.Gly289Arg | Class C0 (GV: 97.85 - GD: 42.00) | Deleterious (score: 0.01) | Disease causing (p-value: 1) | RCV000408960.1 | Very long chain acyl-CoA dehydrogenase deficiency | CM034380 | Very long chain acyl-CoA dehydrogenase deficiency | DM | 0.00009128 | 0.00045500 | rs200788251 |
| YMC-14 | *PCCB* | NM_000532.4 | c.1304A>G | p.Tyr435Cys | Class C55 (GV: 21.61 - GD: 191.71) | Deleterious (score: 0) | Disease causing (p-value: 1) | RCV000012798.24 | Propionic acidaemia | CM022051 | Propionic acidaemia | DM | 0.00004952 | 0.00318200 | rs121964961 |
| YMC-21 | *PCCB* | NM_000532.4 | c.1304A>G | p.Tyr435Cys | Class C55 (GV: 21.61 - GD: 191.71) | Deleterious (score: 0) | Disease causing (p-value: 1) | RCV000012798.24 | Propionic acidaemia | CM022051 | Propionic acidaemia | DM | 0.00004952 | 0.00318200 | rs121964961 |
| YMC-40 | *LRPPRC* | NM_133259.3 | c.4078G>A | p.Ala1360Thr | Class C0 (GV: 105.57 - GD: 26.10) | Deleterious (score: 0.04 | Disease causing (p-value: 1) | (-) | (-) | CM1715653 | Leigh syndrome | DM | 0.00078820 | 0.00884200 | rs147302249 |
| YMC-46 | *GTPBP3* | NM_032620.3 | c.8G>T | p.Arg3Leu | Class C0 (GV: 353.86 - GD: 0.00) | Tolerated (score: 0.08) | Disease causing (p-value: 0.74) | (-) | (-) | CM1413836 | Leigh syndrome | (-) | (-) | 0.00136400 | (-) |
| YMC-56 | *LRPPRC* | NM_133259.3 | c.3430C>T | p.Arg1144Cys | Class C0 (GV: 260.17 - GD: 0.00) | Tolerated (score: 0.15) | Disease causing (p-value: 1) | RCV000376700 | Leigh Syndrome | CM1715652 | Leigh syndrome | DM | 0.00014220 | (-) | rs760016065 |
| YMC-84 | *PCCB* | NM_000532.4 | c.1304A>G | p.Tyr435Cys | Class C55 (GV: 21.61 - GD: 191.71) | Deleterious (score: 0) | Disease causing (p-value: 1) | RCV000012798.24 | Propionic acidaemia | CM022051 | Propionic acidaemia | DM | 0.00004952 | 0.00318200 | rs121964961 |
| YMC-119 | *LRPPRC* | NM_133259.3 | c.4078G>A | p.Ala1360Thr | Class C0 (GV: 105.57 - GD: 26.10) | Deleterious (score: 0.04 | Disease causing (p-value: 1) | (-) | (-) | CM1715653 | Leigh syndrome | DM | 0.00078820 | 0.00884200 | rs147302249 |
| YMC-149 | *LRPPRC* | NM_133259.3 | c.4078G>A | p.Ala1360Thr | Class C0 (GV: 105.57 - GD: 26.10) | Deleterious (score: 0.04 | Disease causing (p-value: 1) | (-) | (-) | CM1715653 | Leigh syndrome | DM | 0.00078820 | 0.00884200 | rs147302249 |
| YMC-193 | *AARS2* | NM_020745.3 | c.609delT | p.Phe203Leufs*36 | (-) | (-) | (-) | (-) | (-) | (-) | (-) | (-) | 0.00000830 | (-) | (-) |
| YMC-194 | *PCCB* | NM_000532.4 | c.1304A>G | p.Tyr435Cys | Class C55 (GV: 21.61 - GD: 191.71) | Deleterious (score: 0) | Disease causing (p-value: 1) | RCV000012798.24 | Propionic acidaemia | CM022051 | Propionic acidaemia | DM | 0.00004952 | 0.00318200 | rs121964961 |

Abbreviations: ACMG, American College of Medical Genetics and Genomics; DCM, dilated cardiomyopathy; DM, disease-causing mutation; ExAC, Exome Aggregation Consortium; HCM, hypertrophic cardiomyopathy; HGMD, human gene mutation database; KRGDB, Korean Reference Genome Database; MAF, minor allele frequency; NM number, National Center for Biotechnology Information (NCBI) reference sequence; dbSNP, database of single nucleotide polymorphism; VUS, variant of uncertain significance

^a^Align GVGD (http://agvgd.iarc.fr/) was used as a nucleotide-conservation prediction algorithm.

^b^ In silico tools including SIFT (http://sift.jcvi.org) and MutationTaster (http://www.mutationtaster.org) were used to assess the predicted impact of missense change

**Table S3.** Mitochondrial DNA mutations upto probably damaging

| Case | Gene | rCRS position | Homo  plasmy | Hetero  plasmy | Patients haplogroup | Variant haplogroup | Frequency GenBank^a^ | PolyPhen2^b^ | SIFT^b^ | PROVEAN^b^ | dbSNP | Associated_disease | Mitomap status | Conservation |
| --- | --- | --- | --- | --- | --- | --- | --- | --- | --- | --- | --- | --- | --- | --- |
| **YMC-10** | ***MT-ATP8*** | **m.8381A>G** | **+** |  | **M7b1a1b** | **(-)** | **0.0002** | **probably_damaging** | **neutral** | **deleterious** | **.** | **MIDD / LVNC cardiomyopathy.assoc,** | **Pathogenic** | **84.44%** |
| YMC-16 | *MT-ATP8* | m.8547T>C | + |  | M7c1a3 | (-) | 0.0003 | probably_damaging | neutral | neutral | . | . | . | 13.33% |
| **YMC-36** | ***MT-TL1*** | **m.3243A>G** |  | **+** | **C1a** | **(-)** | **0.0002** | **.** | **.** | **.** | **.** | **MELAS /MIDD / Cardiac+multi.organ dysfunction** | **Pathogenic** | **97.78%** |
| YMC-36 | *MT-ATP6* | m.9099C>A | + |  | C1a | (-) | 0.0000 | probably_damaging | neutral | neutral | . | . | . | 75.56% |
| YMC-36 | *MT-CYB* | m.15773G>A | + |  | C1a | V2b1 | 0.0010 | probably_damaging | neutral | neutral | . | LHON | Reported^c^ | 100.00% |
| YMC-48 | *MT-CYB* | m.15113A>G | + |  | D4j3 | (-) | 0.0003 | probably_damaging | neutral | neutral | . | . | . | 33.33% |
| YMC-69 | *MT-CO2* | m.7962T>C | + |  | B4f1 | (-) | 0.0001 | probably_damaging | neutral | neutral | rs373420717 | . | . | 20.00% |
| YMC-86 | *MT-ATP6* | m.9182G>A | + |  | A+152+16362 | (-) | 0.0008 | probably_damaging | neutral | neutral | . | . | . | 95.56% |
| YMC-117 | *MT-ATP6* | m.8945T>C | + |  | D4e2a | (-) | 0.0003 | probably_damaging | deleterious | deleterious | . | . | . | 86.67% |
| YMC-124 | *MT-CYB* | m.14954A>G | + |  | D4j | (-) | 0.0002 | probably_damaging | neutral | neutral | . | . | . | 22.22% |
| YMC-134 | *MT-ATP6* | m.9187T>C | + |  | D4b1b | (-) | 0.0000 | probably_damaging | deleterious | deleterious | . | . | . | 100.00% |
| YMC-136 | *MT-CO2* | m.7980A>G | + |  | R11b | (-) | 0.0001 | probably_damaging | neutral | neutral | . | . | . | 28.89% |
| YMC-139 | *MT-ATP6* | m.9182G>A |  | + | D5b1b2 | (-) | 0.0008 | probably_damaging | neutral | neutral | . | . | . | 95.56% |
| YMC-151 | MT-RNR2 | m.3083T>C | + | . | M10a1a1a | (-) | 0.0007 | . | . | . | . | . | . | 55.56% |
| YMC-151 | MT-ND6 | m.14337C>T | + | . | M10a1a1a | (-) | 0.0001 | probably_damaging | neutral | neutral | - | . | . | 64.44% |
| YMC-167 | MT-HV2 | m.227A>T | + | . | M7c1a2a1 | (-) | 0.0001 | . | . | . | . | . | . |  |
| YMC-167 | MT-RNR2 | m.2861A>G |  | + | M7c1a2a1 | (-) | 0.0000 | . | . | . | . | . | . | 82.22% |
| YMC-167 | MT-CYB | m.15038A>G | + | . | M7c1a2a1 | (-) | 0.0006 | possibly_damaging | neutral | neutral | rs202045169 | . | . | 33.33% |
| YMC-183 | MT-ATP6 | m.9025G>A | + | . | D5a2a1b | (-) | 0.0006 | probably_damaging | deleterious | deleterious | rs28681063 | . | . | 100.00% |
| YMC-184 | MT-HV2 | m.65T>C | + | . | B4c1b2a | (-) | 0.0001 | . | . | . | . | . | . |  |
| YMC-184 | MT-CO2 | m.7773C>T | + | . | B4c1b2a | (-) | 0.0001 | benign | neutral | neutral | - | . | . | 86.67% |
| YMC-184 | MT-ATP6 | m.9139G>A |  | + | B4c1b2a | (-) | 0.0008 | probably_damaging | neutral | deleterious | - | LHON | Reported^c^ | 95.56% |
| YMC-188 | MT-ATP6 | m.9179T>C | + | . | A5a3a | (-) | 0.0000 | probably_damaging | neutral | deleterious | - | . | . | 84.44% |
| YMC-198 | MT-CO1 | m.6747T>C |  | + | A1 | (-) | 0.0000 | probably_damaging | neutral | neutral | - | . | . | 93.33% |
| YMC-198 | MT-ATP6 | m.8908T>C | + | . | A1 | (-) | 0.0002 | benign | neutral | neutral | - | . | . | 66.67% |
| YMC-208 | MT-RNR2 | m.3083T>C | + | . | D4b1b | (-) | 0.0006 | . | . | . | . | . | . | 55.56% |
| YMC-208 | MT-NC3 | m.5584A>G | + | . | D4b1b | (-) | 0.0006 | . | . | . | . | . | . | . |
| YMC-208 | MT-ATP6 | m.9187T>C | + | . | D4b1b | (-) | 0.0000 | probably_damaging | deleterious | deleterious | - | . | . | 100.00% |
| YMC-208 | MT-ND5 | m.13153A>G | + | . | D4b1b | (-) | 0.0004 | benign | neutral | neutral | - | . | . | 51.11% |

Abbreviations: DEAF, Maternally inherited deafness or aminoglycoside-induced deafness; dbSNP, database of single nucleotide polymorphism; Homoplasmy,pure mutant mtDNA; Heteroplasmy, mixture of mutant and normal mtDNA; LHON, Leber Hereditary Optic Neuropathy; LVNC, Left ventricular noncompaction; MELAS, Mitochondrial Encephalomyopathy, Lactic Acidosis, and Stroke-like episodes; MIDD, Maternally Inherited Diabetes and Deafness.

^a^The GB frequency data is derived from 45,494 full length (FL) human mitochondrial sequences (>15.4 kbp) from GenBank.

^b^ In silico tools including SIFT (http://sift.jcvi.org), PolyPhen2 (http://genetics.bwh.harvard.edu/pph2/) and PROVEAN (http://provean.jcvi.org/index.php) were used to assess the predicted impact of missense change

^c^Reported" status indicates that one or more publications have considered the mutation as possibly pathologic.

Rows in bold are pathogenic variants in MITOMAP database (https://www.mitomap.org/foswiki/bin/view/MITOMAP/WebHome) and putative pathogenic variants associated with cardiomyopathy.

**Table S4.** Comparisons of cardiac magnetic resonance imaging findings between the patients with and without any sarcomere variants of uncertain significance within patients without any pathogenic or likely pathogenic sarcomere gene variants.

|  | **Sarcomere VUS** (n=32) | **Absence of Sarcomere VUS** (n=60) | ***P*** |
| --- | --- | --- | --- |
| **LVEDV**, mL | 140.0±29.2 | 133.4±27.7 | 0.297 |
| **LVESV**, mL | 49.1±25.1 | 45.8±13.8 | 0.406 |
| **LV mass**, g | 150.2±35.7 | 154.2±53.6 | 0.672 |
| **LV mass index**, g/m^2^ | 83.7±19.6 | 85.3±5.9 | 0.749 |
| **LV mass/volume ratio** | 1.10±0.29 | 1.16±0.33 | 0.352 |
| **LV ejection fraction**, % | 65.7±13.0 | 65.8±7.6 | 0.982 |
| **Segmental^*^ thickness**, mm |  |  |  |
| **Thickness in anteroseptal segments** | 11.9±3.5 | 11.6±3.1 | 0.740 |
| **Thickness in septal segments** | 13.0±4.2 | 12.3±3.3 | 0.388 |
| **Thickness in inferoposterior segments** | 8.8±1.8 | 8.9±1.9 | 0.887 |
| **Thickness in lateral segments** | 8.3±2.0 | 8.8±2.3 | 0.332 |
| **Thickness in apical segments** | 10.2±3.2 | 10.9±4.2 | 0.326 |
| **Late gadolinium enhancement (LGE)** |  |  |  |
| **Presence of LGE in LV,** n (%) | 21 (66) | 34 (57) | 0.504 |
| **Number of LGE segments in LV** | 3.1±3.7 | 2.8±3.3 | 0.711 |
| **% LGE amount of LV** | 8.8±10.6 | 5.8±8.4 | 0.177 |
| **LGE in anteroseptal segments**, % | 23.1±25.4 | 19.4±26.7 | 0.519 |
| **LGE in septal segments**, % | 21.3±25.9 | 16.3±26.4 | 0.392 |
| **LGE in inferoposterior segments**, % | 16.9±27.9 | 13.0±22.3 | 0.500 |
| **LGE in lateral segments**, % | 12.5±24.8 | 14.0±24.5 | 0.782 |
| **LGE in apical segments**, % | 32.0±39.8 | 27.5±36.4 | 0.594 |
| **T2**, ms |  |  |  |
| **T2 average of 16 segments** | 55.4±3.4 | 55.2±2.9 | 0.837 |
| **T2 in anteroseptal segments** | 56.2±3.9 | 56.0±3.0 | 0.841 |
| **T2 in septal segments** | 57.3±4.4 | 57.2±3.4 | 0.900 |
| **T2 in inferoposterior segments** | 54.7±3.5 | 54.9±3.2 | 0.829 |
| **T2 in lateral segments** | 54.0±3.2 | 54.0±3.3 | 0.947 |
| **T2 in apical segments** | 57.5±5.1 | 56.3±3.9 | 0.272 |
| **Native T1**, ms |  |  |  |
| **T1 average of 16 segments** | 1,030.3±46.7 | 1,013.3±49.7 | 0.118 |
| **T1 in anteroseptal segments** | 1,028.5±50.9 | 1,010.7±50.5 | 0.125 |
| **T1 in septal segments** | 1,035.1±51.7 | 1,018.6±47.2 | 0.149 |
| **T1 in inferoposterior segments** | 1,043.9±47.2 | 1,025.4±56.5 | 0.107 |
| **T1 in lateral segments** | 1,023.6±49.8 | 1,007.4±56.0 | 0.170 |
| **T1 in apical segments** | 1,027.3±55.5 | 1,011.7±58.9 | 0.226 |
| **Post T1**, ms |  |  |  |
| **T1 average of 16 segments** | 605.1±70.7 | 608.1±59.3 | 0.839 |
| **T1 in anteroseptal segments** | 599.4±74.5 | 604.3±58.4 | 0.734 |
| **T1 in septal segments** | 604.9±74.4 | 608.9±57.7 | 0.782 |
| **T1 in inferoposterior segments** | 616.2±68.4 | 616.3±61.4 | 0.992 |
| **T1 in lateral segments** | 610.1±66.1 | 611.7±64.9 | 0.912 |
| **T1 in apical segments** | 582.1±74.4 | 590.8±70.0 | 0.584 |
| **Extracellular volume fraction (ECV)**, % |  |  |  |
| **ECV average of 16 segments** | 32.0±4.2 | 31.1±4.4 | 0.341 |
| **ECV in anteroseptal segments** | 32.6±4.3 | 31.4±4.4 | 0.246 |
| **ECV in septal segments** | 32.1±4.4 | 31.0±4.0 | 0.301 |
| **ECV in inferoposterior segments** | 31.2±4.0 | 30.6±4.7 | 0.555 |
| **ECV in lateral segments** | 31.1±4.6 | 30.5±5.3 | 0.612 |
| **ECV in apical segments** | 34.9±5.6 | 33.2±5.4 | 0.191 |

VUS, variant uncertain significance; see abbreviations in table 2.
